# In operando visualization of redox flow battery in membrane-free microfluidic platform

**DOI:** 10.1073/pnas.2114947119

**Published:** 2022-02-23

**Authors:** Hyungjoo Park, Giyun Kwon, Hyomin Lee, Kyunam Lee, Soo Young Park, Ji Eon Kwon, Kisuk Kang, Sung Jae Kim

**Affiliations:** ^a^Department of Electrical and Computer Engineering, Seoul National University, Seoul 08826, Republic of Korea;; ^b^Department of Material Science and Engineering, Seoul National University, Seoul 08826, Republic of Korea;; ^c^Department of Materials Science and Engineering, Research Institute of Advanced Materials, Seoul National University, Seoul 08826, Republic of Korea;; ^d^Department of Chemical & Biological Engineering, Jeju National University, Jeju 63243, Republic of Korea;; ^e^Center for Supramolecular Optoelectronic Materials (CSOM), Seoul National University, Seoul 08826, Korea;; ^f^Functional Composite Materials Research Center, Korea Institute of Science and Technology (KIST), Jeonbuk 55324, Republic of Korea;; ^g^Center for Nanoparticle Research at Institute for Basic Science (IBS), Seoul National University, Seoul 08826, Republic of Korea;; ^h^School of Chemical and Biological Engineering, Institute of Chemical Processes, Seoul National University, Seoul 08826, Republic of Korea;; ^i^SOFT Foundry Institute, Seoul National University, Seoul 08826, Republic of Korea;; ^j^Inter-University Semiconductor Research Center, Seoul National University, Seoul 08826, Republic of Korea

**Keywords:** membrane-free redox flow battery, in operando visualization, multiredox organic molecule, in-depth study, electrochemistry and hydrodynamics

## Abstract

Redox flow batteries (RFBs) are attractive large-scale energy storage techniques, achieving remarkable progress in performance enhancement for the last decades. Nevertheless, an in-depth understanding of the reaction mechanism still remains challenging due to its unique operation mechanism, where electrochemistry and hydrodynamics simultaneously govern battery performance. Thus, to elucidate the precise reactions occurring in RFB systems, an appropriate analysis technique that enables the real-time observation of electrokinetic phenomena is indispensable. Herein, we report in operando visualization and analytical study of RFBs by employing a membrane-free microfluidic platform, that is, a membrane-free microfluidic RFB. Using this platform, the electrokinetic investigations were carried out for the 5,10-bis(2-methoxyethyl)-5,10-dihydrophenazine (BMEPZ) catholyte, which has been recently proposed as a high-performance multiredox organic molecule. Taking advantage of the inherent colorimetric property of BMEPZ, we unravel the intrinsic electrochemical properties in terms of charge and mass transfer kinetics during the multiredox reaction through in operando visualization, which enables theoretical study of physicochemical hydrodynamics in electrochemical systems. Based on insights on the electrokinetic limitations in RFBs, we verify the validity of electrode geometry design that can suppress the range of the depletion region, leading to enhanced cell performance.

## Significance

The current study investigates fundamentals of electrochemical reactions using the membrane-free redox flow battery (RFB) platform with a laminar strategy and colorimetry of multiredox organic molecules. Taking advantage of unique color changes of electrolytes depending on the state of charge, we analyze the electrochemical kinetics of the RFB system in terms of charge and mass transfer. It is verified that a balanced rate of charge and mass transfer significantly affects the battery performance. Furthermore, a classical physicochemical hydrodynamic equation is adopted for scaling analysis of the depletion region deteriorating battery performance. We successfully integrate analytical, numerical, and experimental data for elucidating the depletion region. Based on these fundamental studies, finally, a favorable design is suggested for performance enhancement.

Unprecedented demands for renewable energy resources have become one of the acutest challenges to date, but their intermittent supply is clearly limiting the efficient utilization of these resources (1). Large-scale energy storage systems (ESSs) thus should be paired to mitigate the intermittency for unstoppable delivery of their full promise. Among state-of-the-art ESSs, redox flow batteries (RFBs), using energy-bearing liquid electrolytes, have drawn significant attention because of their unique scalable architecture, with the design flexibility of decoupling power and energy (2). In light of these advantages, extensive research has been conducted for the commercialization of large-scale RFBs, such as vanadium- and zinc/bromine-based RFBs (3). Furthermore, new RFB systems based on various redox couples have been introduced in recent years. In particular, the most recent research has focused on exploiting redox-active organic materials (ROMs) as catholyte/anolyte materials due to their exclusive features of cost-effectiveness, environmental friendliness, and chemical tunability (4, 5). A number of ROMs (e.g., quinone- and tempo-based derivatives) were successfully demonstrated with extraordinary electrochemical performance, which could rival the commercial vanadium- or zinc/bromine-based systems.

Despite the rapid growth of RFB technologies, general tools to probe the reaction mechanisms of these various active materials in flow-dynamic electrochemical cells have not been well established. Considering that the RFB is a dynamic system where electrochemistry and fluid dynamics concurrently govern the performance, advanced analytic platforms that can probe mass and charge transport are indispensable for elucidating the complex electrokinetic phenomena of the redox couples. In conventional lithium battery fields, real-time analytic techniques have been widely employed, which could aid in elucidating the hidden intermediate electrochemical reactions, such as the phase transformation/kinetics of electrode materials by high-speed synchrotron X-ray diffraction, the thermal runaway mechanism of lithium-ion batteries by X-ray tomography, or observing the lithium dendrite growth process in lithium metal electrodes by cryo-transmission electron microscopy analysis, etc. (6–13). While these in situ techniques were valuable to unraveling the solid-state reactions occurring in lithium batteries, the dynamic flow of active liquid-state catholytes/anolytes in RFBs requires new sets of characterization tools. It is because the electrochemical performance of RFBs is dependent on the balancing of the extrinsic flow rate of the active liquids by a mechanical pump and the intrinsic charge-transfer kinetics of the active molecules on the electrode. The interplay of these two, which are not commonly considered in conventional lithium batteries, should be considered in devising the strategy to enhance electrochemical performance; however, it has been largely overlooked to date. Recent attempts to employ in situ characterizations have proven the importance of real-time observation of RFB reactions. Zhao et al. recently succeeded in probing the redox chemistry of quinone-based ROMs in RFBs by in situ NMR metrology, which could unveil the electrolyte decomposition mechanism and quantify the rate of electron transfer (14). Moreover, the crossover of some active materials, such as vanadium- and quinone-based materials, could be directly witnessed by in situ optical spectrophotometry and electron paramagnetic resonance (EPR), respectively (15, 16).

Herein, we introduce in operando visualization of an RFB system using a microfluidic platform that can concomitantly probe the charge and mass transfer of ROMs. The platform made of transparent materials enables real-time observation inside the cell so that the highly coupled electrochemistry and fluid dynamics could be successfully analyzed. In addition, a laminar interface in a microfluidic channel allows battery operation without a membrane; thus the fundamental flow-dynamic study can be systematically performed (17, 18). In our demonstration, we chose to investigate the multiredox organic molecule 5,10-bis(2-methoxyethyl)-5,10-dihydrophenazine (BMEPZ), possessing two stages of color change depending on charge states, which is highly advantageous for optical in operando visualization. In-depth experimental and numerical kinetic studies based on electrokinetics and classical physicochemical hydrodynamics successfully identify the presence of the rate-limiting region and propose an electrode design that can address optimization of the charge and mass transfer kinetics of ROMs (19–21). This in operando visualization tool employing a membrane-free microfluidic cell is expected to aid in elucidating various electrokinetic reactions in dynamic flow-based electrochemical systems.

## Results

### Concept of Microfluidic Membrane-Free RFB (MFRFB).

**Fig. 1.**
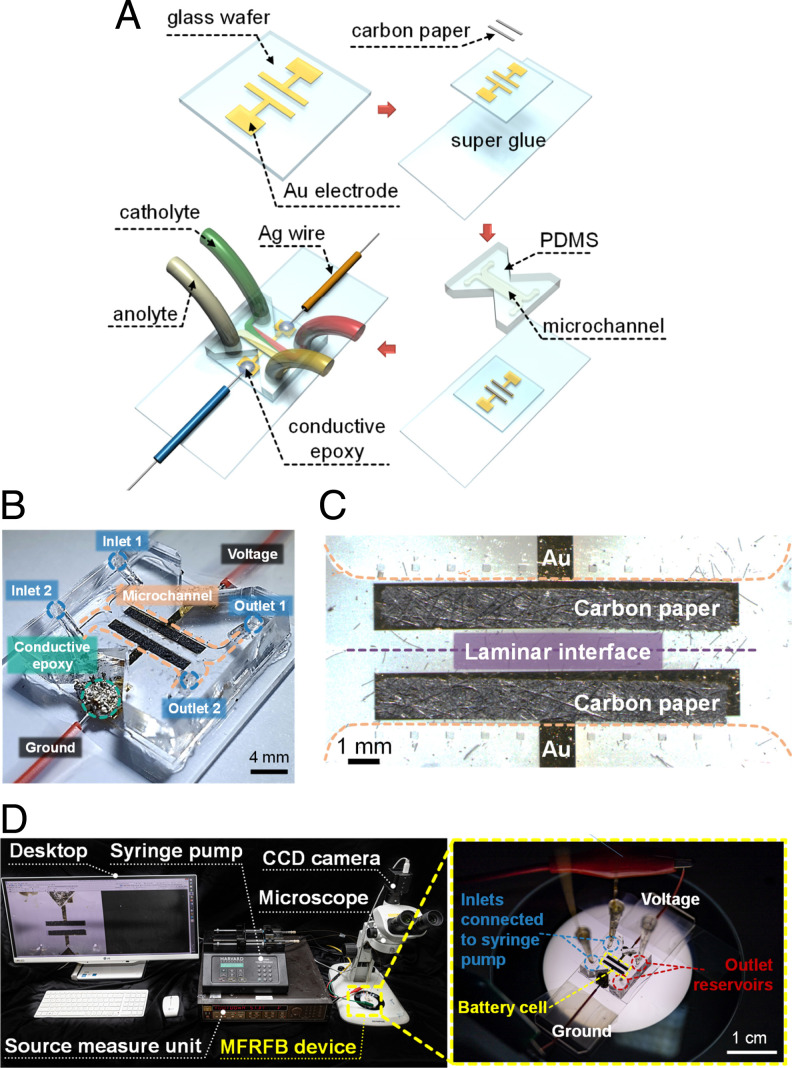
(*A*) The microscale fabrication process of the MFRFB device. (*B*) Photo of an assembled MFRFB device connected to an electrical source; GND, xyz. (*C*) Microscopic view of the observation area for the current study. (*D*) Experimental setup of the in operando visualization experiment. The setup consisted of an MFRFB device on the microscope, a syringe pump, a source measure unit, and a desktop for recording images.

To investigate the intrinsic mass- and charge-transfer reactions occurring in RFBs, a microfluidic platform was employed, which can implement microfluidic manipulation, such as controlling flow rate and tracking of charge states of the catholyte (17, 22). The fabrication process and the assembled device are schematically illustrated in Fig. 1 *A* and *B*, respectively. See *Materials and Methods* for the detailed fabrication processes and setups. In this study, a catholyte through inlet 1 to outlet 1 and an anolyte through inlet 2 to outlet 2 were pumped at the same flow rate (Fig. 1*B*). We intentionally designed the microscale dimension of the fluidic system so that two electrolyte streams form a stable fluidic boundary called a laminar interface, as shown in Fig. 1*C*, which is the region of interest in this work. To obtain laminar flows, the Reynolds number and Peclet number in the current work were coordinated as *Re* < 10 and 800 < *Pe* < 1,700 so that we can neglect inertia of flow. The detailed discussion of the laminar flow formation can be found in *SI Appendix*, Supplementary Note. Since the laminar interface is not ideal in real systems due to unpredictable stimuli from the outside world, crossovers of catholytes and anolytes might inevitably happen in a membrane-free battery system. Nevertheless, when the 9-fluorenone (FL)^−^ or BMEPZ^+^ ions are shuttled, a supplementary charge transfer reaction between FL^−^ and BMEPZ^+^ could occur, which corresponds to a reversible self-discharge reaction. In addition, an irreversible parasitic side reaction is expected not to occur because FL and BMEPZ ions are chemically stable and reversible in the given potential windows, as confirmed in our previous work (22). Furthermore, in order to even avoid this unwanted shuttle, the flow rate was coordinated in the window of high Peclet number (*Pe* > 800) regime, where negligible diffusive mixing is guaranteed. To confirm this, we performed an ultraviolet-visible (UV-vis) test of electrolyte samples at reservoirs to double check that the miscibility of anolyte and catholyte is negligible in the current study (*SI Appendix*, Fig. S1).

However, when it comes to circulation, one must consider accumulation of diffusive mixing of electrolytes. As shown in *SI Appendix*, Fig. S2, we calculated the minimum flow rate as a function of aspect ratio and plotted for different *Pe* numbers. To achieve over 90% of electrolyte utilization during 100 cycles, the key parameters such as flow rate, geometric configuration, and *Pe* need to be highly considered.

As per the application of external current, the electrochemical reaction begins at both electrodes, with the ion exchange for charge balance through the laminar interface instead of the membrane. Since the BMEPZ redox molecule has the colorimetry property, displaying color change depending on state-of-charge (SOC) states, the multiredox reaction of the catholyte can be readily detectable by microscopic observations. The observation was carried using the experimental setup shown in Fig. 1*D*.

### In Operando Visualization of Charge and Mass Transfer in MFRFB.

**Fig. 2.**
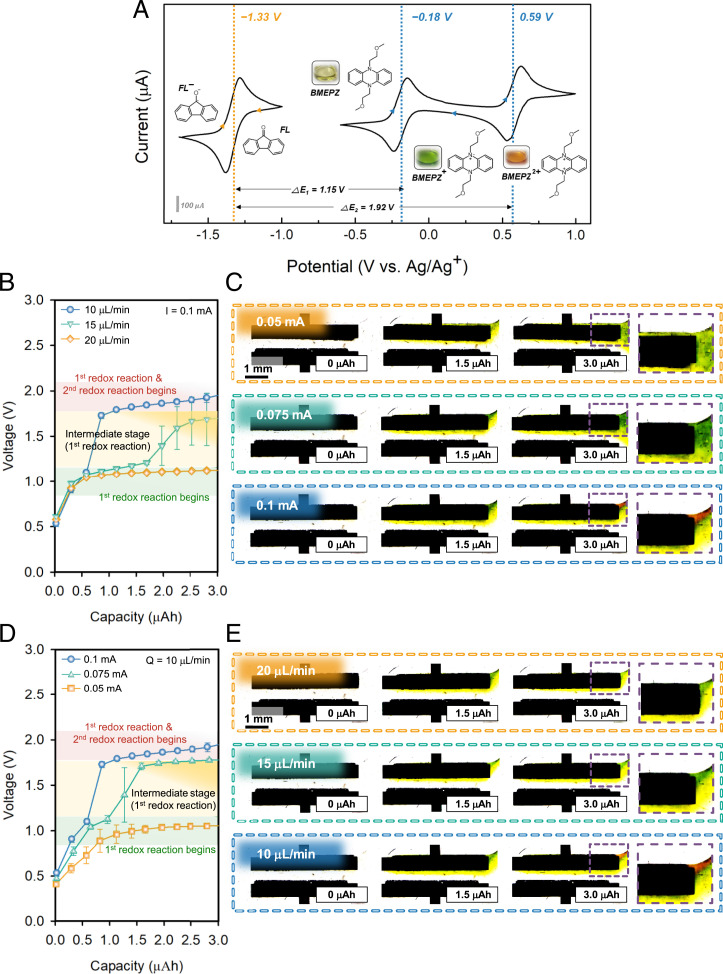
(*A*) The cyclic voltammetry of the redox couple of BMEPZ/FL. (*B*) Voltage and capacity profiles of MFRFB using the redox couple of BMEPZ/FL in terms of charge transfer. (*C*) In operando visualization of the electrochemical reaction in terms of charge transfer. (*D*) Voltage and capacity profiles of the MFRFB using the redox couple of BMEPZ/FL in terms of mass transfer. (*E*) In operando visualization of electrochemical reaction in terms of mass transfer.

Fig. 2*A* shows the cyclic voltammetry of the organic redox couple used in the current study. As previously reported, BMEPZ, which is a soluble multiredox material for catholyte, undergoes two single-electron redox reactions on the diazabutadiene motif (N-C = C-N) at the redox potentials of −0.18 V and 0.59 V versus Ag/Ag^+^ and displays drastic color changes with respect to the charge states (22, 23). The changes in color occur in two distinct stages, 1) from yellow to green during the first oxidation corresponding to BMEPZ^+^ and 2) from green to red becoming BMEPZ^2+^. To demonstrate the full cell, FL was adopted as an active material for anolyte, which does not present apparent color regardless of SOC. FL generally exhibits a single redox reaction at the redox potential of −1.33 V versus Ag/Ag^+^, enabling the two different full-cell voltages of Δ*E*_1_ = 1.15 V and Δ*E*_2_ = 1.92 V for BMEPZ/FL redox couple in RFBs.

Our microfluidic device equipped with the circulation capability (*SI Appendix*, Fig. S2) could independently control the key kinetic factors in the electrochemical reactions of RFBs, that is, the applied current density and the flow rate of catholytes/anolytes. During operation of the cell with various current densities and flow rates, we carefully tracked the color changes of active materials at the electrode through an in operando visualization technique. The systematic alternations of the kinetic parameters were supposed to reveal the migration behavior of the active materials and the electron-transfer rate after they reach the electrodes. Fig. 2 *B* and *C* depicts the electrochemical profiles and corresponding images of the microfluidic cell as a function of the current densities at a fixed flow rate of 10 μL min^−1^. See Video S1 and *SI Appendix*, Fig. S3 for after image processing of in operando visualization. It clearly illustrates that when the redox plateau appears at the cell voltage of 1.0 V with the current rate of 0.05 mA in Fig. 2*B*, the catholyte turns to green in Fig. 2*C*, corresponding to the oxidation reaction of BMEPZ to BMEPZ^+^ (22). In addition, it can be estimated that the concentration of BMEPZ^+^ corresponds to 3.0 mM by comparing the green color to a color library (*SI Appendix*, Fig. S4). As higher current rates are applied at the cell, such as 0.075 mA and 0.1 mA, the plateau voltage gradually elevates close to 2.0 V, skipping the characteristic voltage plateau of BMEPZ to BMEPZ^+^ at 1.0 V. Moreover, the color of the catholyte partially turns into red, indicating the generation of BMEPZ^2+^ before all BMEPZ undergoes single-electron oxidation. It is noteworthy that when the typical voltage plateau of the second redox (BMEPZ^2+^) appears at 2.0 V, the signature of the BMEPZ^+^ (green color) is still dominantly observable near the electrode even though the 1.0 V plateau is significantly shortened or absent, which indicates significantly inefficient cell operation. It implies that the electron-transfer rates exceed the mass-transfer rate at current rates >0.05 mA (e.g., 0.075 or 0.1 mA), causing the partial overpotential and provoking the second electron-redox reactions. However, the low current of 0.05 mA was the suitable electron-transfer rate for the efficient redox reaction at the given mass-transfer rate. Similarly, the demotion of cell voltage during discharge showed similar trends (*SI Appendix*, Fig. S5). Furthermore, we distinctly verified that the apparent cell voltage elevation with the current increase is not simply the results of the overpotential but is accompanied with the second redox reaction of BMEPZ, as evidenced by the red color observed by this in operando visualization technique.

To further investigate the effect of mass transfer, the flow rates of electrolytes were systematically altered to 10, 15, and 20 μL min^−1^ at a fixed current rate of 0.1 mA in Fig. 2 *D* and *E* (Video S2). It manifests that at the high flow rate of 20 μL min^−1^, the supply of fresh ROMs to the electrode is fast enough to satisfy the given electron-transfer rate (0.1 mA), showing the characteristic plateau of the first redox reaction of BMEPZ. This is in contrast to the case of Fig. 2*C* with 0.1 mA, where the second redox reaction was observed. Similarly, as the flow rate gradually decreases, the premature second redox reaction of BMEPZ could be clearly observable with the red electrolyte color as shown in Fig. 2*E*. It indicates that the relative lack of the mass transfer to the electrode leads to the condition for excessive electron transfer. It was additionally noteworthy that the color change to red occurred mostly at the upper side of the cathode, where the reactants are relatively deficient because of the formation of a depletion region. This will be further discussed in the section of numerical study in terms of diffusion and convection. It infers that the consideration of balanced charge- and mass-transfer rates is highly imperative to efficiently utilize the redox-active materials in the RFB cells. Moreover, it was also meaningful that an in operando visualization technique was able to provide direct clues to interpret the electrochemical reactions in RFBs.

### Electrokinetic Analysis of MFRFB for Charge and Mass Transfer.

**Fig. 3.**
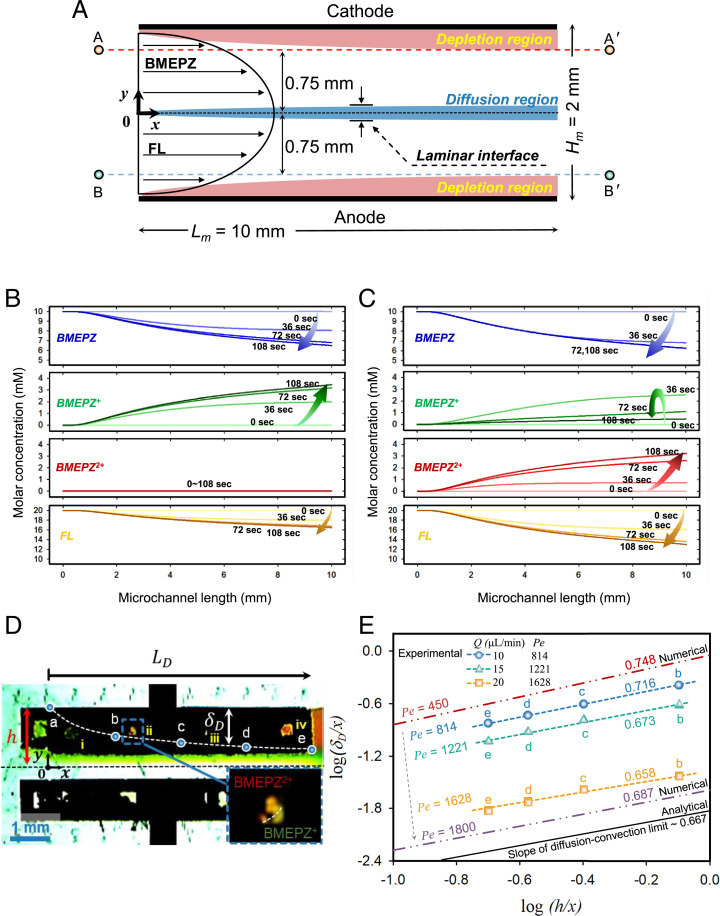
(*A*) Schematic of the MFRFB and theoretical regions that affect battery performance. (*B*) Numerical results of concentration profiles of BMEPZ, BMEPZ^+^, BMEPZ^2+^, and FL^−^ at the low-current density. (*C*) Numerical results of concentration profiles of BMEPZ, BMEPZ^+^, BMEPZ^2+^, and FL^−^ at the high-current density. (*D*) Image of scaling analysis for the concentration boundary layer of BMEPZ^2+^ at a flow rate of 10 μL min^−1^ and a current of 0.14 mA; i, ii, iii, and iv indicate the four windows. (*E*) Comparison of experimental, numerical, and analytical slopes of the concentration boundary layer relation.

Inspired by the observed coupling of electrochemical reaction and fluid kinetics, we attempted to further understand the physicochemical hydrodynamics behind the battery operation and conducted a numerical study of the transport phenomena to support our in operando visualization. Fig. 3*A* schematically illustrates the numerical domain inside the MFRFB cell, although the schematic of the numerical domain is not identical to the real RFB system (Fig. 1*C*). There are two important regions that affect the electrochemical performance, the diffusion region and depletion region where the reactants are relatively deficient (24–27). The diffusion region is formed around the laminar interface due to the diffusive mixing between anolyte and catholyte. However, the depletion region (i.e., reddish stream) is formed near the upper side of the microchannel, where the mass transfer is relatively slow because of the parabolic flow profile of the electrolyte (i.e., near zero at the surface and maximum at the center) as depicted in Fig. 3*A*, leading to faster consumption of reactants at the upper side of the microchannel. These two regions expand in the *y* direction by diffusion and propagate in the *x* direction by convection as the electrolytes flow toward the outlets, while the diffusion could be either accelerated or deaccelerated by electrochemical reactions. It is widely known that with the larger depletion region, the reaction kinetics are generally limited, and the expanded diffusion region also leads to significant crossover of active materials, resulting in deterioration of battery performance.

We conducted two-dimensional (2D) numerical simulation to elucidate the hydrodynamics during the electrochemical reaction by probing changes in the concentration profiles of active materials. The governing equations for momentum and mass transfer were Stokes equations and the diffusion–convection equation, respectively, and the reaction kinetics were included as a boundary condition by the Butler–Volmer relation (*Numerical Model for RFB with Supporting Electrolyte*). Fig. 3 *B* and *C* displays the changes in the concentration profiles of BMEPZ, BMEPZ^+^, BMEPZ^2+^, and FL near the electrode (A-A′ is the line for BMEPZ and B-B′ is the line for FL in Fig. 3*A*) over time as a function of the location in the microchannel for low-current (Fig. 3*B*) and high-current (Fig. 3*C*) density reactions, respectively. Fig. 3*B* shows that the low-current density prevented the second redox reaction of BMEPZ; that is, BMEPZ^2+^ (red color) was not produced until the end of the simulation (108 s), indicating the sufficiently fast charge-transfer reaction, whereas the concentration of BMEPZ^+^ (green color) increased at the expense of BMEPZ (blue line), and FL (yellow line) gradually decreased. This corresponds to 0.05 mA in Fig. 2*C*. However, in the case of the high-current density in Fig. 3*C*, it was found that the concentrations of BMEPZ and FL decreased more drastically, implying that reactants experienced excessive charge transfer at the same given time (i.e., the same flow rate). This harsh condition led to a concurrent reaction of the first and the second redox reactions at the one electrode, where the overall potential was displayed with the second redox value. It is clearly shown that the pristine BMEPZ disappears rapidly upon the electrochemical reaction, and the BMEPZ^+^ was immediately produced due to the first redox reaction within 36 s. However, they are eventually consumed due to the second redox reaction, as indicated with the green arrow in Fig. 3*C*, with the accelerated production of the BMEPZ^2+^ afterward. This corresponds to 0.1 mA in Fig. 2*C*.

Interestingly, the concentration profile of BMEPZ^2+^ in Fig. 3*C*, which can be regarded as the depletion region, resembled a convection–diffusion boundary layer predicted by classical convection–diffusion theory (28, 29). In this regard, we experimentally estimated the thickness of the BMEPZ^2+^ concentration boundary by image mapping of the color changes with a constant current of 0.14 mA and an electrolyte flow rate of 10 μL min^−1^, as shown in Fig. 3*D*. The higher flow rate of electrolyte with fixed constant current cases can be found in *SI Appendix*, Fig. S6. In Fig. 3*D*, four windows (i, ii, iii, and iv) were selectively prepared in the electrodes so that we can directly observe the reddish boundary through the windows. Through the windows, the color gradient could be witnessed (i.e., upper reddish and lower greenish regions in the inset box of Fig. 3*D*), implying the existence of the depletion region, which is coincident with the in operando visualization in Fig. 2 *C* and *E*. Using this windowed device, we could quantitatively investigate the hydrodynamic effects by carefully extracting the approximated concentration boundaries as a function of flow rate (*Q*). By assuming that our MFRFB has a diffusion–convection transportation without electrochemical reactions, we can employ classical diffusion–convection limit relations to characterize the concentration boundary layer versus flow velocity inside the battery cell as follows (19):

[1] $$\frac{\delta_{D}}{x}∼{\left(\frac{h}{x}\right)}^{\frac{2}{3}}{\left(\frac{D}{u_{\max}h}\right)}^{\frac{1}{3}}\equiv {\left(\frac{h}{x}\right)}^{\frac{2}{3}}{\left(Pe\right)}^{-\frac{1}{3}},$$

where, $\delta_{D}$ is thickness of the concentration boundary layer, *D* is the diffusion coefficient, *u*_max_ is maximum velocity of the electrolyte, *h* is height of the battery cell, and *x* is the horizontal distance. *Pe* is Peclet number, which represents the ratio of convective transport to diffusive transport and is defined as *u*_max_*h*/*D*. Based on Eq. **1**, we conducted a scaling analysis. As shown in Fig. 3*E*, the experimental data at the high *Pe* limit (*Pe* = 1,628) and the theoretical prediction by Eq. **1** were remarkably well matched as a slope of 0.658, while the slopes were deviated as 0.716 at the low *Pe* number region (*Pe* = 814). Since the convection is predominant over diffusion and electrochemical reaction at the high *Pe* limit, one could adapt the classical diffusion–convection theory. However, the contribution of the electrochemical reaction, which can steepen the concentration gradient of reactant, becomes significant to the diffusional migration at the low *Pe* number limit (e.g., slope increased as *Pe* decreased). This is why the experimental results at relatively low *Pe* number would have good agreements with numerical simulation, which rigorously considered the electrochemical reactions.

### Enhancing Battery Performance by Redesign of the Electrode Geometry.

**Fig. 4.**
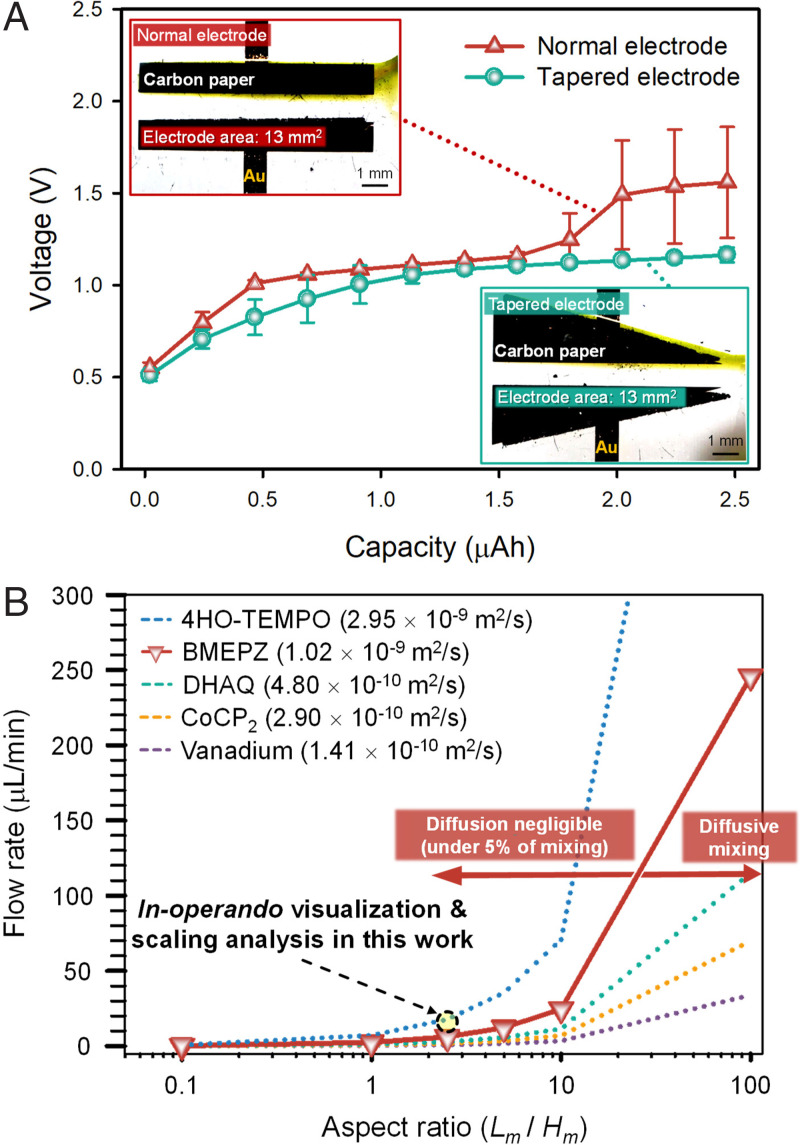
(*A*) Comparison of electrochemical performance with respect to the geometric configuration of the electrode. (*B*) Phase diagram of the required minimum flow rate to make mixing of reactants negligible during one flow depending on the aspect ratio of cells for various active materials in RFBs with corresponding diffusion coefficients; 4HO-TEMPO, BMEPZ, DHAQ, CoCP2, Vanadium.

Two-dimensional numerical simulation and scaling analysis in our current work suggested that the depletion region would be easily developed and hinder the electrochemical redox reaction. Moreover, there are numerical simulations investigating the effect of electrode design (24, 25). Based on our analysis, we compared a tapered electrode (i.e., wider beginning and narrower end) and normal electrode (i.e., straight beginning and end), as shown in the insets of Fig. 4*A*. We rationally designed the tapered electrode considering that the shape of the depletion boundary is roughly proportional to *x*^1/3^ (*x* is horizontal distance). The tapered design was expected 1) to promote the reaction at the wider end of the electrode where a thinner depletion region was formed and 2) to suppress the reaction at the narrower end where there was a thicker depletion region. In this design, even though the surface areas of tapered and normal electrodes are kept identical, the effective electrode area becomes wider in the tapered electrode than in the normal electrode. As shown in the capacity-voltage plot (Fig. 4*A*; *Q* = 10 μL min^−1^ and *I* = 0.1 mA), two distinct forms of evidence could draw the conclusion that the tapered electrode had a superior performance to that of the normal one. First, the overall overpotential was low in almost the entire range of the capacity with the tapered electrode, which meant energy efficiency was higher. Second, and most importantly, the tapered electrode induced the first redox reaction only (i.e., green color), while the second redox reaction was witnessed with the normal electrode (i.e., red color) with the same charge capacity. In other words, the reaction kinetics are more stably and effectively operated with the tapered electrode. Numerical results depending on different electrode geometry also support the experimental results (*SI Appendix*, Fig. S7).

We believe that this kinetic analysis made possible from the in operando visualization would serve as the experimental platform enabling an in-depth study about electrokinetic parameters and an effective means to optimize various types of RFBs. For example, an inevitable diffusive mixing between catholyte and anolyte is a nuisance in a membrane-free (or potentially microporous membrane) platform and thus should be minimized (30–35). Our platform can feasibly suggest how the mixing can be regulated with respect to cell design and flow rate of the catholyte/anolyte in the cell. Fig. 4*B* illustrates the criterion of required minimum flow rates for 5% mixing of the catholyte and anolyte as a function of the aspect ratio of the cell for various active materials having different diffusion coefficients in RFBs; the solid line and dotted line denote the case of BMEPZ and other reported active materials in RFBs, respectively. As the convection of electrolytes needs to dominate over their diffusion for the physical separation of the catholyte and anolyte, the minimum flow rate should increase in proportion to the aspect ratio of the cell, because the extent of diffusion increases as the length of the microchannel. Moreover, as the diffusion coefficient of active material increases, a higher flow rate is required to maintain the minimum mixing of the electrolyte. When less than 5% of mixing is allowed in the battery operation, the cell design with the flow rate should lie in the region of the left side of the solid line (as denoted with diffusion negligible in the plot) for the BMEPZ catholyte. It indicates that this diagram of the correlation between the flow rate and the cell dimension can be utilized in a versatile manner for various active materials with different requirements of the RFB design.

## Discussion

We successfully demonstrated an MFRFB system that can probe the fundamental electrokinetic correlations of multiredox ROMs for RFBs. In operando visualization inside the electrochemical cell was demonstrated to be a promising tool compared to conventional tools, since one could directly observe the electrochemical reaction coupled with hydrodynamics. The changes in redox reactions were clearly observed in real time for the charge- and mass-transfer rates, which are indispensable to elucidate the electrokinetic phenomena in RFBs. Moreover, combined study of 2D numerical simulation and scaling analysis of the concentration boundary layer presented the clue for the bottleneck regions that can deteriorate battery performance during electrochemical reaction in a convective diffusion system. Based on the insights of the electrokinetic limitations in RFBs, enhanced battery performance was achieved through the redesign of electrode geometry, having the tapered geometry with a larger effective electrode area. These investigations present the importance of the in operando analysis technique for better understanding of dynamic RFB systems.

## Materials and Methods

### Microfabrication of Microfluidic MFRFB.

The MFRFB system requires several microscale fabrications for inducing laminar flow during electrochemical reactions (36–45). As shown in Fig. 1*A*, the metal deposition process (Au lift-off process) was conducted on the surface of a 4-inch glass wafer for electrode patterning. The thickness of Au/Ti electrodes was 1,100 Å (Au, 1,000 Å; Ti, 100 Å). Ti was previously deposited as an adhesion layer between the glass and gold electrode. The glass wafer was diced into a square (20 mm × 20 mm), and it was bonded onto a slide glass (2.5 cm × 7.5 cm; Daihan Scientific) using Scotch super glue (3M). Then, precut porous carbon felts (200 ± 20 μm thick; XF30A, TOYOBO) were overlaid onto the electrodes.

A polydimethylsiloxane (PDMS) block (Slygard 184 silicone elastomer kit, Dow Corning) as a microfluidic RFB cell was molded by predefined silicon master. The master had a microfluidic channel pattern of two bifurcated inlets and outlets. Briefly, the PDMS solution was mixed with a curing agent at a ratio of 10:1 and degassed for 1 h in a vacuum chamber. The degassed solution was poured into the master and was cured in the oven for 4 h at 75 °C. Then, the PDMS block was detached from the master, and triangular pieces were cut from the block for connecting an external current source. The length and height of the microchannel was 10 and 1 mm, respectively, and the depth of the microchannel was 200 μm so that carbon paper on an Au electrode could be sandwiched between the glass wafer and PDMS. The two inlets and two outlets were punched by biopsy punch (BF-15F, Kai Medical) to create 1.5-mm-diameter holes. Au electrodes were deposited on the glass by E-gun evaporator. After metal deposition, glass and PDMS block were irreversibly bonded using oxygen plasma (CUTE-MP, Femto Science, Korea) treatment. The process of fabrication and imaging of assembled devices is shown in Fig. 1 *A* and *B*.

### Chemical Preparation.

Acetonitrile (MeCN) and FL were purchased from Sigma-Aldrich and used as received. Bis(trifluoromethane)sulfonimide lithium salt (LiTFSI) was purchased from TCI Chemicals and dried under vacuum at 180 °C for 24 h to remove moisture. BMEPZ was synthesized following a previously reported synthetic route (23).

### General Experimental Setups.

For the electrochemical measurements, the electrolytes were prepared and evaluated in an Ar-filled glove box under an inert atmosphere (<0.5 ppm O_2_, H_2_O), except the one flow charging experiment. Pipette tips as electrolyte reservoirs were inserted into each inlet and outlet of the PDMS block. Ag wires were glued onto the electrode using conductive epoxy (silver conductive epoxy 8330S-21G, MG Chemicals).

### One Flow Charging Experiment.

The molar concentration of catholyte for in operando visualization of multiredox experiments was 10 mM, and constant currents of 0.05, 0.075, and 0.1 mA were applied to the microfluidic RFB by a source measure unit (Keithley 238) via Ag electrodes connected to the Au electrodes. The electrolytes were infused by a syringe pump (Harvard Apparatus PHD 2000 syringe pump) from inlets to outlets. A 1-min resting time was given for fully filling the microchannel and build-up for the laminar interface of electrolytes. After resting time, a constant current was applied for 120 s. For the measurement of voltage and capacity characteristic, a customized LabVIEW program was used. For reliability, one flow charge experiment was conducted five times for identical devices and conditions. Images and video files of in operando visualizations were captured by a stereomicroscope system (Olympus, SZ61) and CellSens program.

### Circulation Experiment.

For the circulation experiment, a peristaltic pump (LabV6) and constant current mode using a battery test system (WBCS 3000, WonA Tech) were used. Symmetric redox couple of BMEPZ and BMEPZ^+^ (each 1 mM) in the supporting electrolyte of 0.5 M LiTFSI in MeCN (0.5 mL) was used with a flow rate of 1 mL min^−1^. The volume of electrolytes was 0.7 mL.

### CV Curve Measurement.

 Cyclic voltammetry (CV) curves of BMEPZ and FL (10 mM each) were captured using the supporting electrolytes of 0.5 M LiTFSI in MeCN. A three-electrode system (Pt counter electrode, Ag/AgNO_3_ reference electrode, and glassy carbon working electrode) was employed with a scan rate of 100 mV s^−1^. For the UV-vis spectroscopy analysis to verify diffusive mixing at the laminar interface of electrolytes, absorption spectra of the inlet and outlet catholytes (diluted in MeCN, 5% [vol/vol]) were obtained using a UV-vis spectrometer (Agilent Technologies, Cary 5000) with an optical glass cuvette (Quartzl Hellma).

### Numerical Model for RFB with Supporting Electrolyte.

In the actual microfluidic flow battery of this work, the electrolytes used were 10 × 10^−3^ M BMEPZ as a catholyte, 20 mM FL as an anolyte, and 0.5 M LiTFSI as a supporting electrolyte. The supporting electrolyte was an inert salt with a concentration much greater than other electrolyte components. Due to the supporting electrolyte, the transport phenomena of catholyte, anolyte, and other related derivatives can be described by the convection–diffusion equation (46, 47). In the battery systems, there were five kinds of electrolytic species, which were involved in electrochemical reactions: FL, FL^–^, BMEPZ, BMEPZ^+^, and BMEPZ^2+^. Thus, we solved the convection–diffusion equations for each electrolytic species and the continuity equation and the Stokes equations for flow field (**u**) and pressure (*p*) inside the numerical domain, as shown in *SI Appendix*, Fig. S8. For a convenience, each electrolyte concentration was denoted as *c*_FL_ for FL, *c*_FL1_ for FL^–^, *c*_BM_ for BMEPZ, *c*_BM1_ for BMEPZ^+^, and *c*_BM2_ for BMEPZ^2+^, respectively. Detailed formulations were given as detailed below.

The convection–diffusion equation for each species was given by

[2] $$\frac{\partial c_{t}}{\partial t}=-\nabla \;\cdot [D_{i\;}\nabla c_{i}+c_{i}u]\text{,}$$

where *c_i_* is the concentration of the *i*th species, *t* is the time, *D_i_* is the diffusivity of the *i*th species, and **u** is the flow field. Note that the electrochemical reactions were only on the electrode surface so that any reaction terms were omitted in the above equation. Instead, the electrochemical reactions were described by the appropriate boundary conditions. The flow field and pressure field were described by the continuity equation and the Stokes equations:

[3] ∇⋅u=0,

[4] $$\rho \frac{\partial u}{\partial t}=-\nabla p+\mu \nabla^{2}u,$$

where *ρ* is the fluid viscosity, *p* is the pressure, and *μ* is the fluid viscosity.

The boundary conditions on inlet 1, as depicted in *SI Appendix*, Fig. S6, were

[5] $$c_{\text{BM}}=10\;\text{mM,}$$

[6] $$c_{i}=0\;\;\text{for}\;i\neq \text{BM,}$$

[7] $$\int_{0}^{H}u_{x}dy=UH,$$

where *H* is the half-height of the numerical domain, *u_x_* is the *x*-directional flow field, and *U* is the specific value of the averaged flow velocity. Eqs. **5** and **6** mean that only BMEPZ was introduced through inlet 1. Eq. **7** is a constraint for averaged value. Collaborating with no-slip condition at inert wall and the electrode surface, the flow field becomes the Hagen–Poiseuille flow. Similarly, on inlet 2,

[8] $$c_{\text{FL}}=20\;\text{mM,}$$

[9] $$c_{i}=0\;\text{for}\;i\neq \text{FL,}$$

[10] $$\int_{-H}^{0}u_{x}dy=UH.$$

On inert wall, no penetration condition for each electrolyte species and no-slip condition for fluid flow were imposed:

[11] $$n\cdot \nabla c_{i}=0,$$

[12] u=0,

where **n** is the outward normal vector. On the upper electrode, there were BMEPZ-related electrochemical reactions. Such reactions caused effective electrolyte flux through the boundary. Dealing with the Butler–Volmer equation, the boundary conditions were

[13] $$-n\cdot \nabla c_{\text{BM}}=-\frac{i_{\text{BM}}}{F}\left[\frac{c_{\text{BM}}}{c_{\text{ref}}}\exp\left(\frac{F\eta_{\text{BM}}}{2RT}\right)-\frac{c_{\text{BM}1}}{c_{\text{ref}}}\exp\left(-\frac{F\eta_{\text{BM}}}{2RT}\right)\right],$$

[14] $$-n\cdot \nabla c_{\text{BM}1}=-n\cdot \nabla c_{\text{BM}}+n\cdot \nabla c_{\text{BM}2},$$

[15] $$-n\cdot \nabla c_{\text{BM}}{}_{2}=-\frac{i_{\text{BM}1}}{F}\left[\frac{c_{\text{BM}1}}{c_{\text{ref}}}\exp\left(\frac{F\eta_{\text{BM}1}}{2RT}\right)-\frac{c_{\text{BM}2}}{c_{\text{ref}}}\exp\left(-\frac{F\eta_{\text{BM}1}}{2RT}\right)\right],$$

[16] $$n\cdot \nabla c_{\text{FL}}=n\cdot \nabla c_{\text{FL}1}=0,$$

[17] u=0,

where *i*_BM_ and *i*_BM1_ are the exchange current density for related electrochemical reactions, of which values are 2.849 A m^−2^ and 6.681 A m^−2^, *F* is the Faraday constant, *c*_ref_ is the experimental concentration of the exchange current measurement (1 mM), *η*_BM_ and *η*_BM1_ are the overpotential for electrolytic species, *R* is the gas constant, and *T* is the absolute temperature. The definitions of overpotential were

[18] $$\eta_{\text{BM}}=V_{\text{BM}}-E_{\text{BM}}^{\text{eq}},$$

[19] $$\eta_{\text{BM}1}=V_{\text{BM}}-E_{\text{BM1}}^{\text{eq}},$$

where *V*_BM_ is the electrical potential at the upper electrode, and $E_{\text{BM}}^{\text{eq}}$ and $E_{\text{BM1}}^{\text{eq}}$ are the equilibrium potentials, of which the values are –0.18 V and 0.59 V. Since the actual experiments were done in constant current mode, following additional constraint on the upper electrode should be solved simultaneously.

[20] $$\int_{0}^{L}n\cdot \nabla c_{\text{BM}1}+2n\cdot \nabla c_{\text{BM}2}dx=-\frac{i_{\mathrm{app}}L}{F},$$

where *i_app_* is the applied current density as a constant. Similar to the upper electrode, the boundary conditions on the bottom electrode were given by

[21] $$-n\cdot \nabla c_{\text{FL}}=-\frac{i_{\text{FL}}}{F}\left[\frac{c_{\text{FL}}}{c_{\text{ref}}}\exp\left(\frac{F\eta_{\text{FL}}}{2RT}\right)-\frac{c_{\text{FL1}}}{c_{\text{ref}}}\exp\left(-\frac{F\eta_{\text{FL}}}{2RT}\right)\right],$$

[22] $$-n\cdot \nabla c_{\text{BM}1}=n\cdot \nabla c_{\text{FL}},$$

[23] $$\eta_{\text{FL}}=V_{\text{FL}}-E_{\text{FL}}^{\text{eq}},$$

[24] $$\int_{0}^{L}n\cdot \nabla c_{\text{BM1}}dx=-\frac{i_{\mathrm{app}}L}{F},$$

[25] u=0,

where *i*_FL_ is the exchange current density, of which the value is 2.849 A m^−2^, *V*_FL_ is the electrical potential at electrode 2, and $E_{\text{FL}}^{\text{eq}}$ is the equilibrium potential, of which the value is –1.33 V. The duration of the multiredox reaction was 108 s. Simulation parameters are summarized in *SI Appendix*, Table S1.

It was confirmed in our previous work that BMEPZ underwent two single-electron redox reactions at different redox potentials, which means that two-step oxidations of BMEPZ to BMEPZ^+^ and BMEPZ^+^ to BMEPZ^2+^ occur during the charging process (22). In addition, based on high material utilization and great stability, the redox couple underwent redox reaction without any parasitic side reaction. In this manner, we assumed that the Butler–Volmer equation can be applied in our numerical model because the redox reactions of redox couple are based on elementary reaction without any submechanism. In the numerical model, reaction mechanisms of BMEPZ and FL are multistep and single step of one electron reaction, respectively.

### Numerical Simulation Method.

Numerical simulation was performed by COMSOL Multiphysics 5.4 using a personal computer with an AMD Ryzen 7 3700X 8-Core Processor and 16 gigabytes of memory. A general form partial differential equation module, global ordinary differential equations (ODEs) module, creeping flow module, and time-dependent solver in COMSOL Multiphysics were employed to compute all of the governing equations and boundary conditions. Calculation time of the numerical simulation was within 3 min. For appropriate numerical process, a nonuniform mesh structure was introduced after proper convergence test.

### Data Processing of Numerical Simulation for Concentration Profiles of Electrolyte.

According to a convection–diffusion equation of hydrodynamics and previous research that addresses the depletion and diffusion region inside the MFRFB system, we assumed that the concentration profiles of BMEPZ, BMEPZ^+^, BMEPZ^2+^, and FL^−^ have parabolic shapes as electrolytes shuttle toward the outlets as they simultaneously undergo electrochemical reaction. To verify our assumption, we conducted numerical simulations for concentration profiles of electrolytes near electrodes as a function of time and microchannel length. First, we appointed A (0, 0.75 mm), A′ (10 mm, 0.75 mm), B (0, −0.75 mm), and B′ (10 mm, −0.75 mm) in the numerical domain. Second, we obtained one-dimensional concentration profiles of electrolytes as a function of microchannel length by exporting line data in the postprocessing module in COMSOL. In addition, the total duration of numerical simulation was 108 s. Along the time, data points at 0, 36, 72, and 108 s were selected since they showed representative changes of concentration profiles. As a result, we extracted the concentration profiles of BMEPZ, BMEPZ^+^, BMEPZ^2+^, and FL^−^ for 0, 36, 72, and 108 s, respectively (BMEPZ, BMEPZ^+^, and BMEPZ^2+^ at line A-A′ and FL^−^ at line B-B′). Finally, we overlaid all of the plots to show time-evolving changes of the concentration profile during electrochemical reactions (Fig. 3 *B* and *C*).

## Supplementary Material

Supplementary File

Supplementary File

Supplementary File

## Data Availability

Image, real time video file and data set of voltage-capacity characteristics in terms of charge and mass transfer of in operando visualization have been deposited in Zenodo, the open-access repository (https://zenodo.org/record/5992944, https://zenodo.org/record/5992654 and https://zenodo.org/record/5992891). All other study data are included in the article and/or supporting information.
