# Supplementary material for: In operando visualization of redox flow battery in membrane-free microfluidic platform
Source: Proc Natl Acad Sci U S A. 2022 Feb 23;119(9):e2114947119. doi: 10.1073/pnas.2114947119 (PMC8892322; doi:10.1073/pnas.2114947119)
Supplement: Supplementary File [file pnas.2114947119.sapp.pdf]

PNAS
www.pnas.org

Supplementary Information for

In-operando visualization of redox flow battery in membrane-free microfluidic platform

Hyungjoo Park†, Giyun Kwon†, Hyomin Lee†, Kyunam Lee, Soo Young Park, Ji Eon Kwon, Kisuk Kang*, Sung Jae Kim*

Corresponding authors : Kisuk Kang, Sung Jae Kim

Email: Kisuk Kang(matlgen1@snu.ac.kr) , Sung Jae Kim(gates@snu.ac.kr)

This PDF file includes:

Supplementary Note

Figures S1 to S8

Tables S1

SI references

Other supplementary materials for this manuscript include the following:

Movies to S1 to S2

Laminar flow

When fluid flows in channel or pipe, the flows can be characterized by Reynolds number(1). Reynolds number is a dimension-less number that concerns various properties of fluid itself and system where fluid flows. *Re* number can be expressed as follows:

$$Re = \frac{\rho U L}{\mu}$$

Where μ the dynamic viscosity of the fluid, ρ is the density of the fluid, U is the mean velocity of the fluid and L is the characteristic length of system. The value of dimensionless number varies with material properties and system scale. If the value of Reynolds number is less than 2000, then flow can be characterized as laminar flow. On the other hand, if the value of Reynolds number is greater than 4000, then flow can be characterized as turbulent flow.

In this study, the flow rates of BMEPZ and FL are adapted to make laminar flows along the microchannel. As a result, the ion transport can be achieved without membrane since the laminar interface function as a physical barrier for crossover of reactant(2).

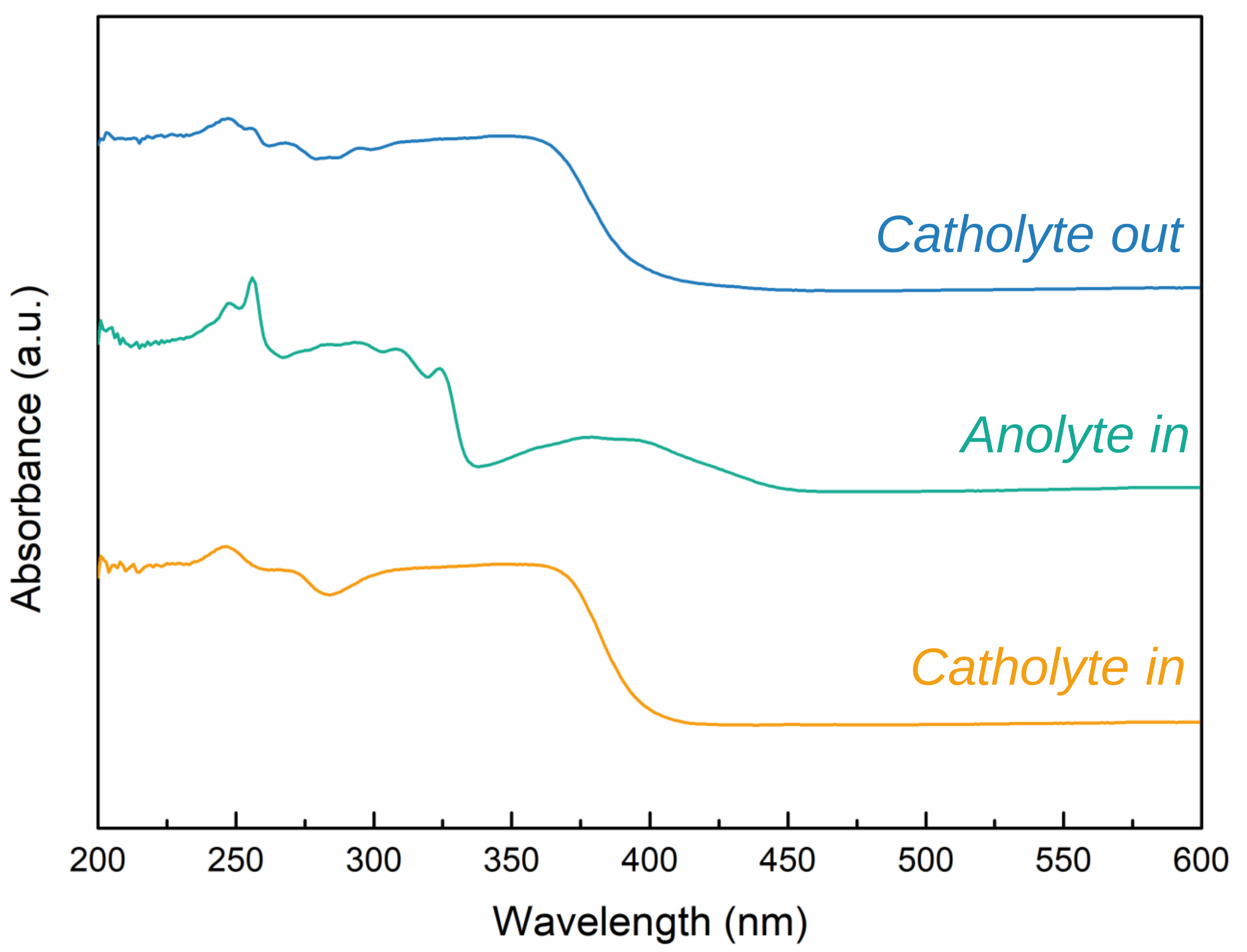

Fig. S1. UV-vis test of electrolyte during one flow experiment. The data were collected at room temperature using 10 mM BMEPZ and FL.

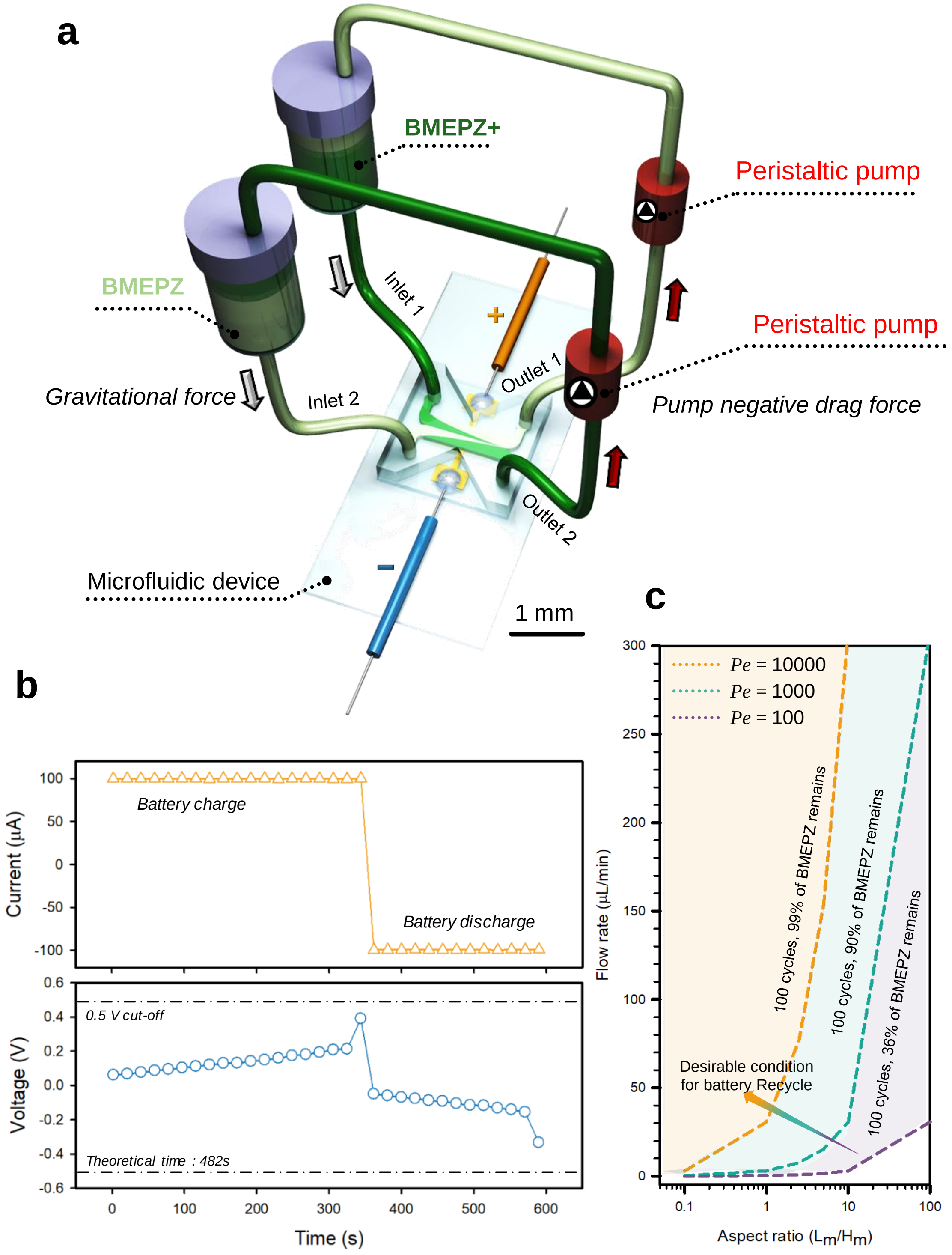

Fig. S2. Circulation experiment and cycle estimation of MFRFB system. a, Schematic of circulating MFRFB system. **b,** Voltage *vs.* time profile using symmetric cell. **c**, Flow rate profile for electrolyte utilization during 100 cycles depending on the cell geometry.

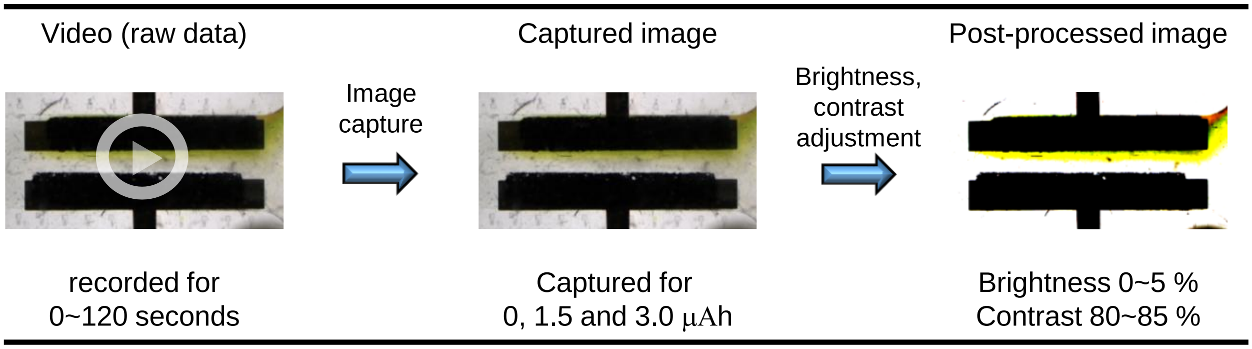

Fig. S3. Post image processing of *in-operando* visualization. As shown in Fig. S3, we recorded *in-operando* visualization of electrochemical reaction under microscopic observation, while critical parameters such as flow rate and current were adjusted. The duration of video was 2 minutes for all experimental conditions and we captured images when cell capacity reaches 0, 1.5 and 3.0 μAh for Fig. 2C and Fig. 2E. In the next step, we extracted the length of reddish color (*i.e*. color of $BMEPZ^{2+}$) by adjusting contrast 85% enhance and brightness 5% enhance. In case of scaling analysis (Fig. 3D and 3E), the adjustments were contrast 80% and brightness 0%.

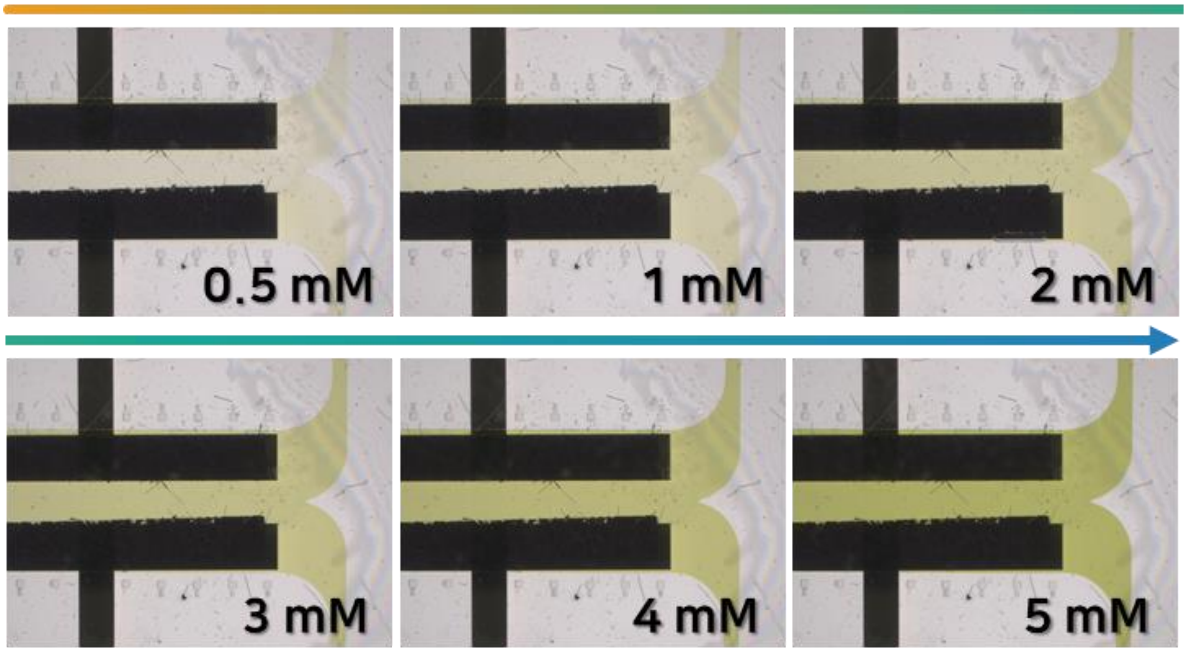

Fig. S4. Color library of BMEPZ^{+} from the concentration. The range if concentration of BMEPZ^{+} was 0.5 mM to 5.0 mM for quantitative analysis.

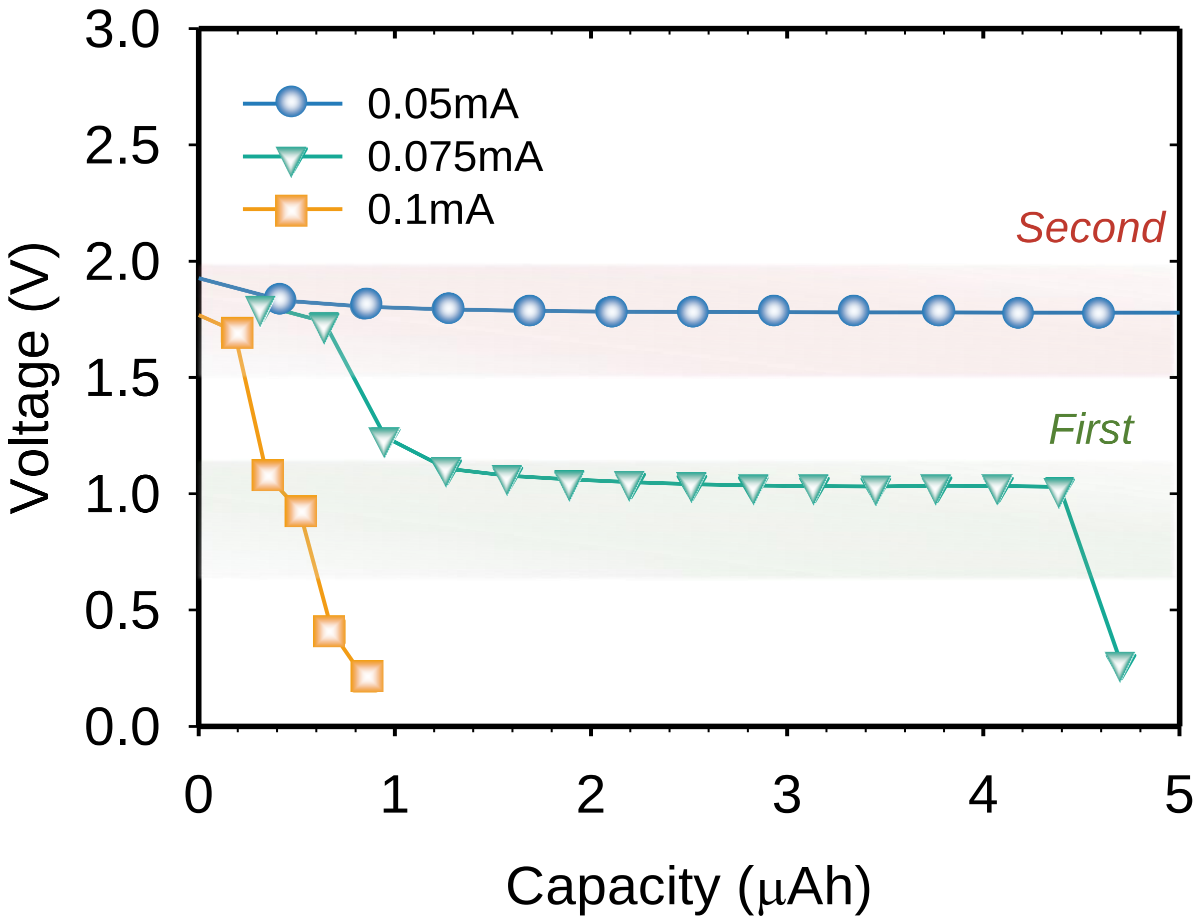

Fig. S5. Discharge experiment of one flow through experiment. The experiment was performed inside the glove box using 10.0 mM BMEPZ and FL. The flow rates of electrolyte was 10 μL min^{-1}.

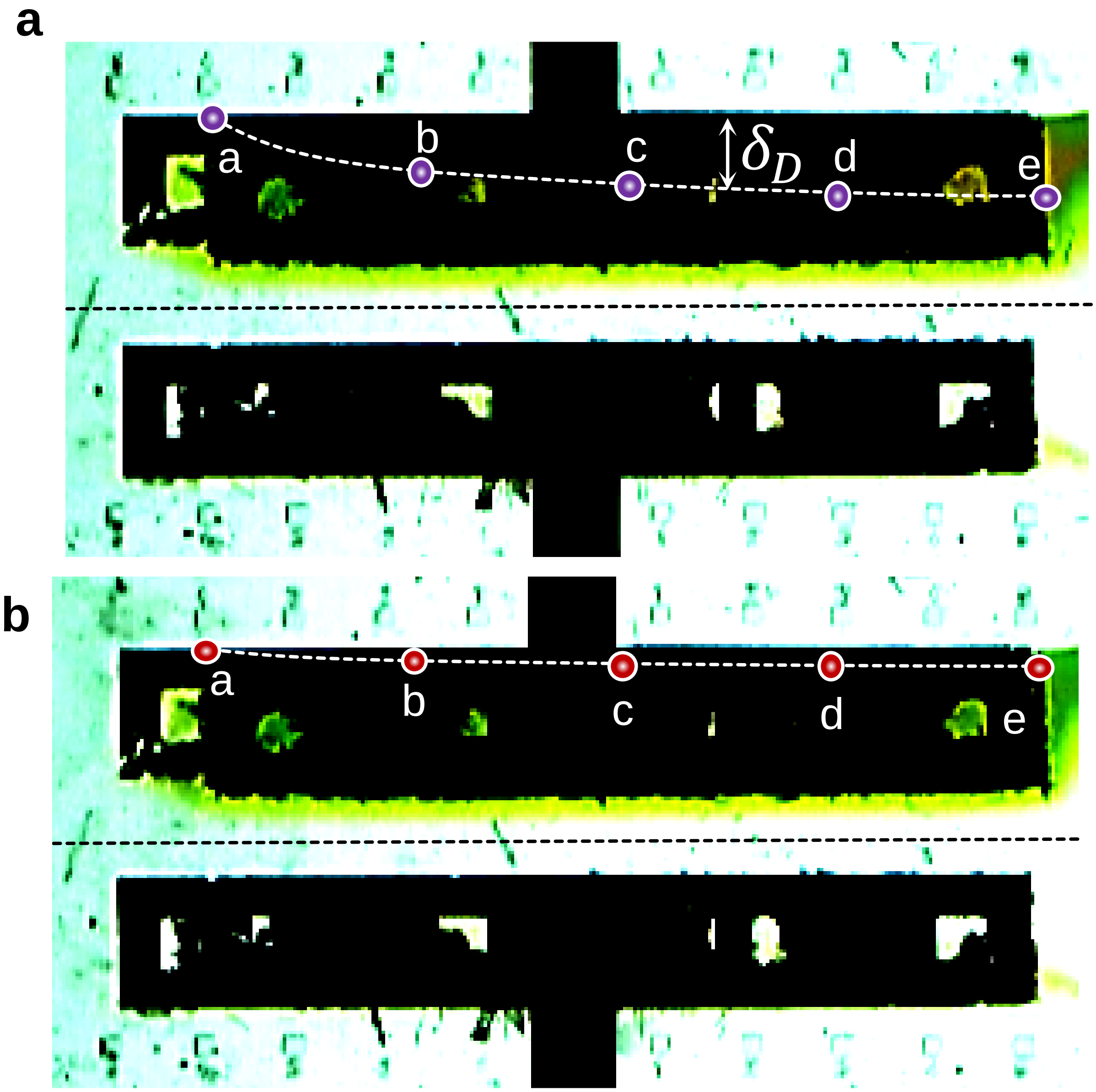

Fig. S6. *In-operando* visualization of scaling analysis for high *Pe* case. a, The flow rate of electrolytes was 15 μL min^{-1} and *Pe* was 1221. **b**, The flow rate of electrolytes was 20 μL min^{-1} and *Pe* was 1628. For both cases, the applied constant current was 0.14 mA.

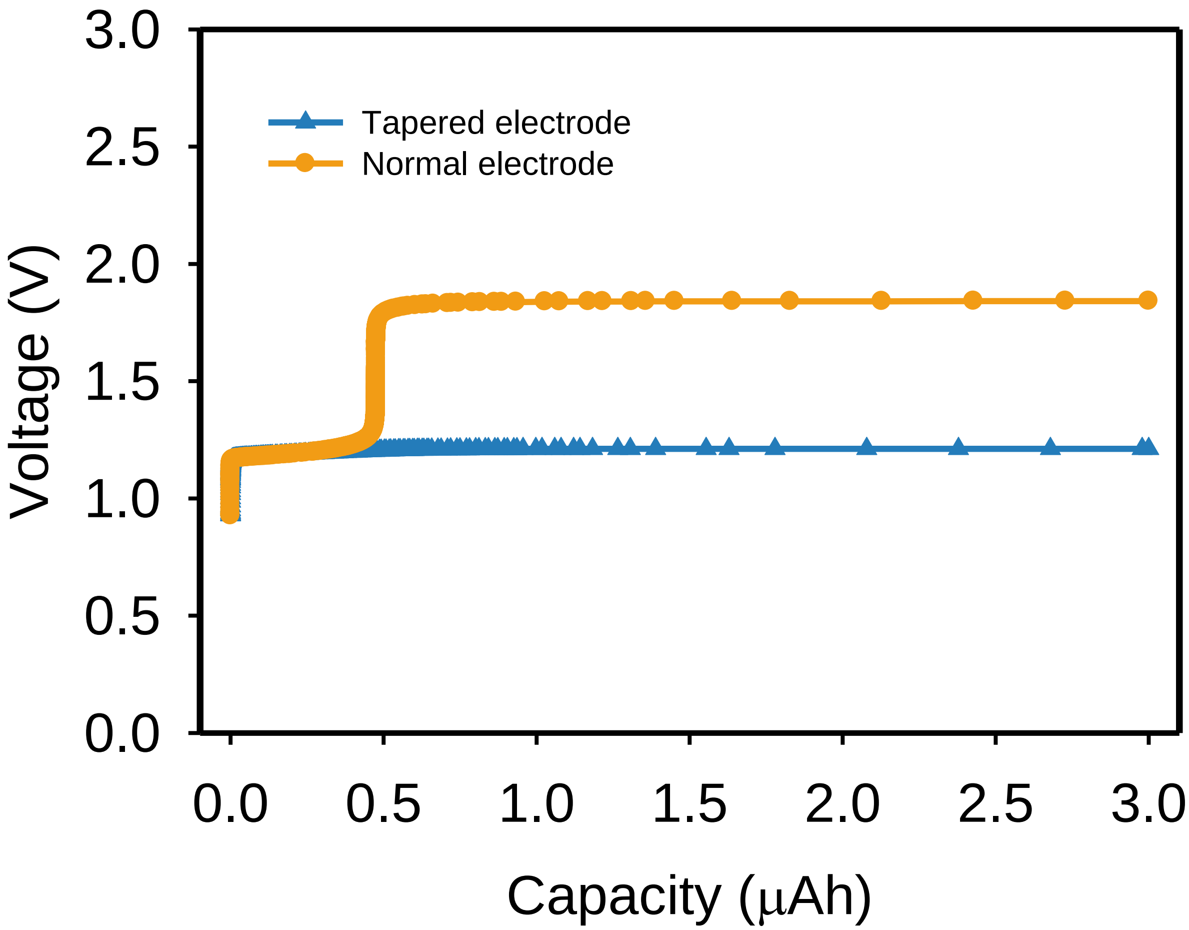

Fig. S7 Numerical results of voltage and capacity profile depending on the electrode geometry comparison. Applied current and flow rate was 0.1 mA and 10 μL min^{-1} respectively.

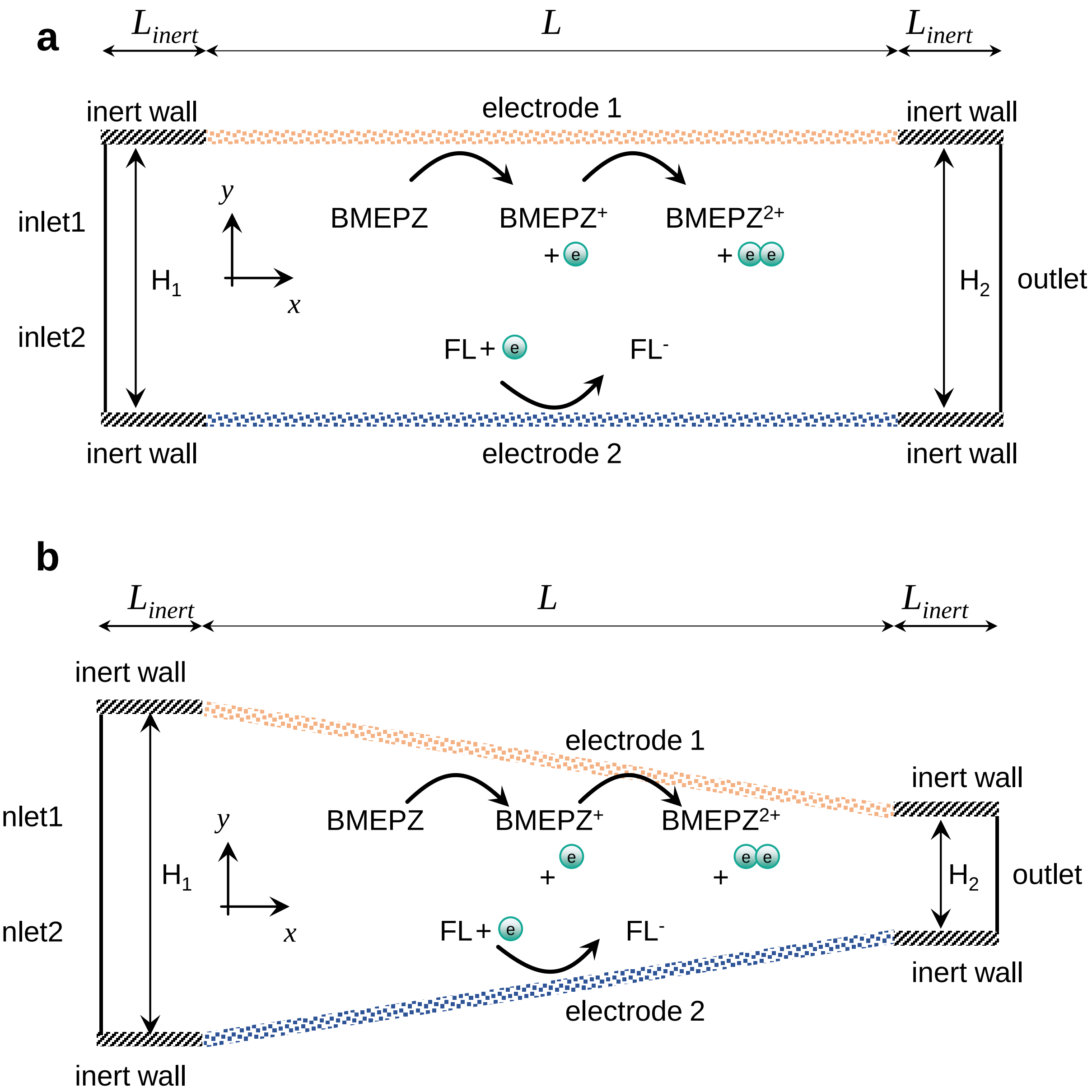

Fig. S8. Numerical domain of MFRFB system. a, Numerical domain for *in-operando* visualization and scaling analysis. **b**, Numercial domain for tapered electrode. The depth of microchannel was 180 μm and area of electrode was 13 mm^2.

Parameter	Value	Description
H_1	2 mm	Height of inlet in normal electrode
	3 mm	Height of inlet in tapered electrode
	4 mm	Height of inlet in scaling analysis
H_2	2 mm	Height of outlet in normal electrode
	1 mm	Height of outlet in tapered electrode
	4 mm	Height of outlet in scaling analysis
L	10 mm	Length of electrode
L_{inert}	1 mm	Length of inert wall
ρ	1000 kg m^{-3}	Fluid density
μ	0.001 Pa s	Fluid viscosity
U	4.63×10^{-4} m s^{-1}<br>9.26×10^{-4} m s^{-1}<br>2.32×10^{-4} m s^{-1}	Averaged flow velocity for *in-operando* visualization<br>Average flow velocity for scaling analysis (Pe = 1800)<br>Average flow velocity for scaling analysis (Pe = 450)
T	300 K	Temperature
D_{BM}	1.02×10^{-9} m^2 s^{-1}	BMEPZ diffusivity(3)
D_{BM1}	1.12×10^{-9} m^2 s^{-1}	BMEPZ$^+$ diffusivity(3)
D_{BM2}	1.12×10^{-9} m^2 s^{-1}	BMEPZ^{2+} diffusivity(4)
D_{FL}	1.05×10^{-9} m^2 s^{-1}	FL diffusivity(5)
D_{FL1}	1.05×10^{-9} m^2 s^{-1}	FL$^-$ diffusivity(4)
i_{BM}	2.85 A m^{-2}	Exchange current density for BMEPZ/BMEPZ$^+$ redox reaction(3)
i_{BM1}	6.68 A m^{-2}	Exchange current density for BMEPZ$^+$/BMEPZ^{2+} redox reaction(3)
i_{FL}	2.85 A m^{-2}	Exchange current density for FL/FL$^-$ redox reaction(5)
i_{app}	7.69 A m^{-2}<br>3.85 A m^{-2}<br>6.15 A m^{-2}	Applied current density for *in-operando* visualization (I = 0.1 mA) & tapered electrode<br>Applied current density for *in-operando* visualization (I = 0.05 mA)<br>Applied current density for scaling analysis
E_{BM}^{eq}	–0.18 V	Equilibrium potential for BMEPZ/BMEPZ$^+$ redox reaction(3)
E_{BM1}^{eq}	0.59 V	Equilibrium potential for BMEPZ$^+$/BMEPZ^{2+} redox reaction(3)
E_{FL}^{eq}	–1.33 V	Equilibrium potential for FL/FL$^-$ redox reaction(5)

Table S1. Physicochemical parameters used for electrochemical reaction. Diffusion coefficients of BMEPZ, BMEPZ$^+$ and FL were calculated through the rotating disk electrode (RDE) test. Moreover, in numerical model, we applied Stokes-Einstein relation(4) to assume that diffusion coefficients of BMEPZ^{2+} and FL$^-$ are identical to those of BMEPZ$^+$ and FL, respectively.

SI References

1. L. G. Leal, *Laminar flow and convective transport processes : scaling principles and asymptotic analysis*, Butterworth-Heinemann series in chemical engineering (Butterworth-Heinemann, Boston, 1992), pp. xviii, 740 p.
2. M. A. Goulet, E. Kjeang, Reactant recirculation in electrochemical co-laminar flow cells. *Electrochim Acta* **140**, 217-224 (2014).
3. G. Kwon *et al.*, Bio-inspired Molecular Redesign of a Multi-redox Catholyte for High-Energy Non-aqueous Organic Redox Flow Batteries. *Chem-Us* **5**, 2642-2656 (2019).
4. R. F. Probstein, *Physicochemical hydrodynamics : an introduction* (Wiley, New York, ed. 2nd, 1994), pp. xv, 400 p.
5. G. Kwon *et al.*, Multi-redox Molecule for High-Energy Redox Flow Batteries. *Joule* **2**, 1771-1782 (2018).
